# Promising Bioactivity of Vitamin B_1_-Au Nanocluster: Structure, Enhanced Antioxidant Behavior, and Serum Protein Interaction

**DOI:** 10.3390/antiox12040874

**Published:** 2023-04-03

**Authors:** Ditta Ungor, Gyöngyi Gombár, Ádám Juhász, Gergely F. Samu, Edit Csapó

**Affiliations:** 1 MTA-SZTE Lendület “Momentum” Noble Metal Nanostructures Research Group, University of Szeged, Rerrich B. sqr. 1, H-6720 Szeged, Hungary; 2 Interdisciplinary Excellence Center, Department of Physical Chemistry and Materials Science, University of Szeged, Rerrich B. sqr. 1, H-6720 Szeged, Hungary

**Keywords:** thiamine, gold nanocluster, ORAC, ABTS assay, bovine serum albumin binding

## Abstract

In the current work, we first present a simple synthesis method for the preparation of novel Vitamin-B_1_-stabilized few-atomic gold nanoclusters with few atomic layers. The formed nanostructure contains ca. eight Au atoms and shows intensive blue emissions at 450 nm. The absolute quantum yield is 3%. The average lifetime is in the nanosecond range and three main components are separated and assigned to the metal–metal and ligand–metal charge transfers. Based on the structural characterization, the formed clusters contain Au in zero oxidation state, and Vitamin B_1_ stabilizes the metal cores via the coordination of pyrimidine-N. The antioxidant property of the Au nanoclusters is more prominent than that of the pure Vitamin B_1_, which is confirmed by two different colorimetric assays. For the investigation into their potential bioactivity, interactions with bovine serum albumin were carried out and quantified. The determined stoichiometry indicates a self-catalyzed binding, which is almost the same value based on the fluorometric and calorimetric measurements. The calculated thermodynamic parameters verify the spontaneous bond of the clusters along the protein chain by hydrogen bonds and electrostatic interactions.

## 1. Introduction

In recent years, the biomedical applications of noble metal nanostructures, especially gold (Au), silver (Ag), and copper (Cu), have been of interest to researchers. These have several advantages, such as chemical inertness and tunable optical or surface properties. There are many syntheses that can produce particles with different sizes and morphologies, but for most of them, several other components can also be identified in the resulting suspension. Therefore, the purification and additional surface functionalization of the formed nano-objects are necessary to satisfy strict medical requirements. As a result, biocompatible syntheses offer us an outstanding opportunity to simultaneously reduce the metal ions and stabilize/functionalize the formed particles with only one biomolecule (e.g., proteins, nucleotides, amino acids) [1,2]. The character of this molecule, which predominantly contains nitrogen- or sulfur-containing donor groups, has a large effect on the size of the formed particles, therefore also influencing the optical features. In the case of the application of small molar ratios between the ligand and metal ions, the classical plasmonic particle is synthesized [3]. In contrast, noble metal nanoclusters (NCs) with molecular-like optical properties can be prepared when the reducing ligands are present in an excessive amount relative to the metal ions [4].

Due to the biocompatible shell on NCs, several articles can be found related to their potential application in biomedical fields. Thanks to their environment-sensitive luminescence, they are prominent raw materials for biosensors and light emitters [5]. The detection of biologically important molecules (e.g., glutathione [6], dopamine [7], folic acid [8], *L*-kynurenine [9]), pH [10], and intracellular temperature [11] have been previously studied in several articles. Moreover, they have been successfully applied as theragnostic agents. For example, Zheng and co-workers examined the potential application of mercaptobenzoic acid-, cysteine-, and cysteamine-stabilized Au NCs as a potential antimicrobial agent thanks to their more effective production of harmful reactive oxygen species (ROS) [12] in different bacteria. In their article, S. Jindal and P. Gopinath clearly demonstrated the success of an anti-proliferative agent, a chitosan–Ag nanocluster/4-phenylbutyrate hydrogel, as a histone deacetylase inhibitor for breast cancer cells [13]. For the inhibition of neurodegenerative diseases, Li and co-workers have produced several works focused on the potential anti-amyloid usability of noble metal NCs for the treatment of brain-centered hazards [14].

Due to fast and unhealthy daily routines, stress, and chemicals, several types of ROS (peroxynitrite/ONOO●/, nitric oxide/NO●/, peroxyl radical/ROO●/, superoxide radical/O_2_●^−^/, etc.) can be formed in living organisms, which can cause dysfunction and damage the tissues by facilitating cell death [15,16]. Apart from the generally used antioxidant lipid-based vesicles [17,18], niosomes [19], gels [20], and composite materials [21], ultra-small NCs are potential devices to prevent these issues because their composition is easily tuned by the selection of the appropriate metal and surface ligand to precisely design smart biomimetic materials [22]. Based on the above-mentioned utilization of few-atomic NCs with few atomic layers, antioxidant and enzyme-like applications should be a priority in future research.

In this present work, a one-pot synthesis of blue-emitting Vitamin B_1_-stabilized gold nanoclusters (B_1_-Au NCs) is demonstrated for the first time due to the possible antioxidant application of this hybrid nanostructure. Furthermore, the experimental results for the exact optical and structural characterizations of their potential bioactivity are also presented. It is well known that the Vitamin B molecular family shows an intensive inhibition capacity against ROS; therefore, antioxidant behavior was measured by standard 2,2′-azino-bis(3-ethylbenzothiazoline-6-sulfonic acid) (ABTS) and Oxygen Radical Antioxidant Capacity (ORAC) assays, and protein interactions with bovine serum albumin (BSA) were also investigated and interpreted.

## 2. Materials and Methods

### 2.1. Chemicals

To synthesize blue-emitting B_1_-Au NCs, thiamine hydrochloride (Vitamin B_1_; C_12_H_17_ClN_4_OS·HCl; ≥99.9%; Sigma, Budapest, Hungary), hydrogen tetrachloroaurate(III) hydrate (HAuCl_4_ × H_2_O; 99.9% (metal basis); Sigma, Budapest Hungary), and sodium hydroxide (NaOH; 99%; Molar, Budapest, Hungary) were used. During the antioxidant measurements, 2,2′-azino-bis(3-ethylbenzothiazoline-6-sulfonic acid) diammonium salt (ABTS; C_18_H_24_N_6_O_6_S_4_; ≥98%; Alfa Aesar, Ward Hill, MA, USA), potassium persulfate (K_2_S_2_O_8_; 99.99%; Sigma, Budapest, Hungary), 2,2′-azobis(2-methylpropionamidine) dihydrochloride (AAPH; [=NC(CH_3_)_2_C(=NH)NH_2_]_2_ × 2 HCl; 97%; Sigma, Budapest, Hungary), 6-Hydroxy-2,5,7,8-tetramethylchromane-2-carboxylic acid (Trolox; C_14_H_18_O_4_; 97%; Sigma, Budapest, Hungary), and fluorescein sodium salt (C_20_H_10_Na_2_O_5_; analytical standard; VWR, Debrecen, Hungary) were selected. The buffers and the protein solutions were made by using bovine serum albumin (BSA; lyophilized powder, essentially globulin free; ≥99%; Sigma, Budapest, Hungary), hydrochloric acid (HCl; 37%; Molar, Budapest, Hungary), sodium dihydrogen phosphate 2-hydrate (NaH_2_PO_4_ × 2 H_2_O; 99%; Molar, Budapest, Hungary), disodium hydrogen phosphate dodecahydrate (Na_2_HPO_4_ × 12 H_2_O; ≥99.99%; Molar, Budapest, Hungary), and sodium chloride (NaCl; 99.98%; Molar, Budapest, Hungary). The reagents were used without further purification. The fresh stock solutions were prepared with Milli-Q (Merck Millipore, Darmstadt, Germany) ultrapure water (conductivity: 18.2 MΩ·cm). For the purification, a Pur-A-LyzerTM dialysis kit (Sigma, Budapest, Hungary) with a 1 kDa cut-off value was chosen.

### 2.2. Preparation Protocol of B_1_-Au NCs

For the synthesis of blue-emitting NCs, a B_1_:[AuCl_4_]^−^/25:1 molar ratio was applied, while the final concentration of the Au in the dispersion was 1.0 mM, and the Vitamin B_1_ concentration was 25 mM. In the first step of the synthesis, an adequate amount of the ligand and metal solutions was mixed in ultrapure water. The pH of the reaction mixture was adjusted to pH = 3.5 by using 0.1 M NaOH. After 3 min of stirring, 1 mL of 0.01 M HAuCl_4_ solution was pipetted into the reaction mixture, and then the solution was incubated at 25.0 ± 0.1 °C. After 24 h, the intensive yellow color of the [AuCl_4_]^−^ ions disappeared, and the solution turned pale brownish. A few larger aggregates were formed at the end of the synthesis, which were removed from the sample by centrifugation at 13,000 rpm for 30 min, and the supernatant was further used. The unnecessary synthesis components and the unreacted salts were removed by dialysis for 1 h in a Pur-A-LyzerTM Mega 1000 dialysis kit (MWCO = 1 kDa, Sigma, Budapest, Hungary).

### 2.3. Antioxidant Measurements

For the ABTS and ORAC assays, the stock and working solutions were prepared according to Ref. [23]. For ABTS test, the sample solutions were prepared by mixing 200.0 μL ABTS working solution and 10.0 μL sample solution, which contained the Vitamin B_1_ or the B_1_-Au NCs with a suitable (the same) ligand concentration. The samples were kept in dark for 5 min at 25 °C, then they were diluted to 3 mL and the absorbance of the diluted samples was registered at 734 nm. The inhibition ratio was calculated by the following, Equation (1) [23]:

(1) $$Inhibition\;\%=\left(\frac{A_{c}-A_{s}}{A_{c}}\right)\times 100$$

where the *A_c_* and *A_s_* are the absorbance at 734 nm of the control and sample after 5 min, respectively. The antioxidant behavior was evaluated by the calculation of *IC*_50_ value, where the inhibition extent reached 50%. The samples were investigated in the 5–100 μM concentration range.

In the case of the ORAC test, the fluorescence spectra of fluorescein dye were registered between 500 and 650 nm, in which the emission maxima were located at 500 nm, and the excitation wavelength was 485 nm. An adequate volume of antioxidant material and 78 μL of fluorescein solution (c = 1 μM) were pipetted in phosphate-buffered saline (PBS, c_NaCl_ = 0.15 M, pH = 7.4). The incubation took place at 37 °C for 15 min in the dark. After the incubation, 100 μL of 200 mM AAPH solution was also added to start the reaction. Before the measurements, the volume of the samples was completed with a 2 mL buffer to reach the appropriate fluorescence signal. The hydrophilic Vitamin E derivative Trolox molecule served as a reference. To compare and quantify the activity, the ORAC value [24] was determined by the following formula (Equation (2)):

(2) $$ORAC\;value=\frac{c_{Trolox}\times Net_{AUC\;of\;sample}\times k}{Net_{AUC\;of\;Trolox}}$$

where the *c_Trolox_* is 50 μM, and the *Net_AUC of sample_* and *Net_AUC **of Trolox**_* values are the net areas under kinetic curves for the 50 μM B_1_ content in the sample and 50 μM in the Trolox solution. Each Net_AUC_ value was calculated after the blank correction by the integration of the time-dependent decay curves. The ***k*** is the dilution factor of the sample, which was 4-fold in our case.

### 2.4. Experimental Conditions for the Investigation of Protein Interaction by Spectrofluorometry and Isotherm Titration Calorimetry

For the optical measurements, the solutions were mixed in PBS buffer. In the case of *L*-tryptophane (*Trp*) quenching, the BSA concentration was fixed at 0.5 mM, while the concentration of the NCs was varied between 0 and 1 mM. The samples were stirred for 2 min at 25 °C. The excitation and emission wavelengths were 280 and 348 nm, respectively. For the calorimetric studies, a room temperature of 25 °C was also selected, and the measurements were carried out with a computer-controlled instrument. The sample cell of the calorimeter with 1.4 mL volume was filled with 0.1 mM concentration of Vitamin B_1_ or B_1_-Au NCs, while the syringe contained 280 μL BSA solution in 1.5 mM concentration. The titration speed was 8 μL/step/5 min. For the calculation of the thermodynamic parameters, the one- and two-site binding models were selected. The theoretical background of the evaluation can be found in the Supporting Information section.

### 2.5. Instruments

Fluorescence spectra were registered on the ABL&E JASCO FP-8500 spectrofluorometer in a standard 1 cm quartz cuvette. The measurement was carried out by using 395 nm as excitation wavelength, 2.5–2.5 nm bandwidths, 1 nm resolution, and 200 nm/min scan speed. For the calculation of the absolute internal quantum yield (QY %), the same instrument was used, which was equipped with the ABL&E JASCO ILF-835 integrating sphere. For the calibration, WI light source (ABL&E JASCO ESC-842) was applied; thus, other references were not needed. The evaluation of the spectra was completed in the ABL&E JASCO SpectraManager 2.0 software. The fluorescence lifetime of the NCs was determined by time-correlated single-photon counting (TCSPC) measurements on a Horiba DeltaFlex apparatus using a 1 cm optical length. A DeltaDiode pulsed laser (λ_laser_ = 371 nm) served as an excitation light source. The emitted light was detected at 450 nm with a 6 nm slit. The number of counts on the peak channel was 10,000, and for the instrument response function (IRF), standard LUDOX^®^ SiO_2_ colloids (Horiba) were applied. The lifetime components were calculated by exponential fitting with χ^2^ goodness values of the registered decay curves in the EZTime program from Horiba. For the determination of the binding energies in the main elements in the B_1_-Au NCs, X-ray photoelectron spectra were registered in survey scan (40 eV pass energy) and high-resolution modes (20 eV pass energy). The spectra were collected by a SPECS device equipped with a PHOIBOS 150MCD9 hemispherical analyzer. The sample was deposited on a 0.5 mm thick titanium foil (Sigma) by multistep cyclic freeze-drying. The Al K_α_ X-ray source was operated at 200 W power and the carbon 1s peak (284.80 eV) was used for charge referencing. Spectra were evaluated by CasaXPS software. To identify the possible coordination between the stabilizing Vitamin B_1_ and Au metal cores, Fourier-transformed infrared spectroscopy (FT-IR) was used based on measurement with a JASCO FT/IR-4700 instrument equipped with an ATR PRO ONE single-reflection accessory. The spectra were collected in the range of 500 to 4000 cm^−1^ by 128 interferograms with 1 cm^−1^ resolution on lyophilized powder samples. For the reference thiamine spectra, the powder was prepared at the same concentrations and similar pH to those of the cluster dispersions. The stability of the fluorescent nano-objects was studied on a Malvern Zetasizer NanoZS ZEN 4003 apparatus equipped with a He-Ne laser (λ = 633 nm) at 25 ± 0.1 °C. The ionic strength was regulated by 0.1 M NaCl, and the hydrodynamic diameters (d_H_) were calculated by the Smoluchowski model. The secondary structure of the BSA before and after the addition of NCs was investigated by circular dichroism (CD). For this purpose, ABL&E JASCO J-1100 CD spectrometer was applied, equipped a high-energy Xenon lamp (450 W) and a water-cooled ABL&E JASCO PTC-514 Peltier sample holder. The measurements were carried out at 25 °C in a 1 cm quartz cuvette with 200 nm/min scan speed and 1 nm resolution. The spectra were determined by the Secondary Structure Estimation module of the ABL&E JASCO SpectraManager 2.0 software. The thermodynamic characterization of the BSA/NCs complex system’s formation was investigated by isotherm titration calorimetry using MicroCal VP-ITC power-compensation microcalorimeter equipped with MicroCal 7.1 Origin^®^-based software.

## 3. Results and Discussion

### 3.1. Structural Characterization of the Fluorescent B_1_-Au NCs

The optimization of the synthetic process was carried out to find the ideal parameters for production of nanosized objects with the highest fluorescence intensity. For this purpose, the applied molar ratio between the Vitamin B_1_ and AuCl_4_^−^ ions, the pH of the reaction mixture, the concentration of the precursor metal salt, the temperature, and the synthesis time were examined in individual samples. The final parameters were selected based on the highest emission yield of the samples and the measured data are shown in Figure S1. After the optimization process, it was found that the most fluorescent product was prepared by using a B_1_:[AuCl_4_]^−^/25:1 molar ratio with a 1.0 mM initial aurate ion concentration (Figure S1a,b). The best fluorescent response (as seen in (Figure S1c)) was achieved by a reaction mixture with an initial pH = 3.5. Under more acidic conditions, the pyrimidine-N1 of the thiamine is still protonated [25], which might facilitate the direct coordination of gold(III) ions to the pyrimidine ring of the Vitamin B_1_ molecule via electrostatic interactions. In the case of the deprotonated pseudobase thiamine formed at pH = 4–9, the positive charge is only on the thiazole-N3 nitrogen, which is not able to coordinate to the precursor [AuCl_4_]^−^; thus, this form is not favorable for cluster formation. Under alkaline conditions, the formation of the thiochrome form is the dominant species, which also shows the formation of nanohybrid systems with low fluorescent yields. For the further optimization process, the temperature and the synthesis time were also investigated (Figure S1d,e), in which the samples prepared at 25 °C for 24 h with the previously optimized parameters had superior emissive properties. Before the exact structural characterization, purification via 13,000 rpm centrifugation for 30 min and dialysis were performed to remove the larger aggregates, the unreacted metal salt, and the excess vitamin B_1_. After the purification, the final metal concentration was c_Au_ = 0.75 mM, as measured by inductively coupled plasma mass spectrometry (ICP-MS), while the B_1_ concentration (c_B1_ = 17 mM) was determined based on spectrophotometric data; the final molar ratio was ca. B_1_:Au/22.5:1 in the product, which refers to the total concentrations of the t.

**Figure 1:**
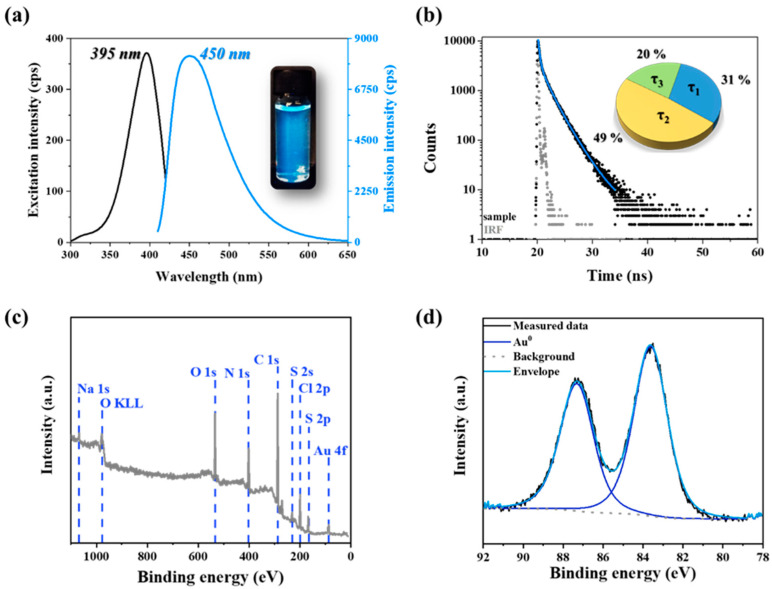
(**a**) The excitation and emission spectra of the B_1_-Au NCs with the photo of the sample under UV lamp (λ_max_ = 365 nm). (**b**) The typical fluorescence decay curve of the B_1_-Au NCs with the distribution percentage of the main lifetime components (λ_ex_ = 371 nm). (**c**) The survey scan and (**d**) the XPS spectra of the Au content in B_1_-Au NCs.

After purification, optical and structural characterizations of the synthesized fluorescent nanomaterial were carried out. As Figure 1a clearly shows, the prepared B_1_-stabilized Au nanohybrid system showed intensive blue emissions under a UV lamp. Based on the steady-state spectrofluorometric measurements, the excitation and the emission maxima were found at 395 and 450 nm, respectively. The absolute internal quantum yield was 3.1 ± 0.2%. The determined optical characteristics refer to the formation of few-atomic NCs and ca. eight Au atoms in the primer cores [26,27]. We determined the average lifetime (**τ**) and the main lifetime components of the prepared system. As can be seen in Figure 1b, the fluorescence lifetime of the Au-containing nano-object is in the nanosecond range, which can indicate the presence of ultra-small metal cores [28]. In the case of the biomolecule-stabilized few-atomic NCs, two to three main τ components can predominantly be separated from the nanosecond-range average lifetime, in which case the shorter components are only metal- or size-dependent, while the longer components show the ligand-dependent charge transfer [29]. By fitting the decay curves, three main lifetime components (τ_1_–τ_3_) can be identified. The τ_1_ is 1.26 ± 0.38 ns (31%), which is in good agreement with the previously published data on the charge transfer to the oxidized form of Vitamin B_1_ (thiochrome) [30,31]. This component can form by metal reduction during the synthesis (Scheme S1), and it is presumably on the surface via *π···π* stacking to take part in the stabilization of the final metal cores [32]. The τ_2_ is 2.46 ± 0.13 ns, which contributes the largest amount (ca. 49%) to the total fluorescence of the sample. Based on the literature data, the τ_2_ belongs to the ligand-to-metal charge transfers. In contrast, the shortest component (τ_3_ = 0.24 ± 0.01, 20%) can be assigned to the metal–metal transitions in the cluster cores.

To determine the surface chemical composition, the XPS spectra were recorded in the case of the powder samples. On the survey scan (Figure 1c), the presence of O, N, C, S, and Cl from the stabilizing ligand are observable, together with the presence of Au from the metal cores. The Au 4f region can be fitted with an asymmetric line shape that is characteristic of metals (Figure 1d). The determined binding energies of the 4f_5/2_ and 4f_7/2_ core-level transitions are located at 87.3 and 83.6 eV, respectively, which shows the presence of metallic Au. The slight negative shift to the 4f_7/2_ of the bulk Au (84.0 eV) might be the result of the ultra-small size of the metal cores with strong ligand–metal electron-donating interactions [33], which was also previously shown in the results of the fluorescence lifetime measurements. The other regions on the XP spectra (Figure S2) indicate the presence of the thiamine molecule on the surface of the Au metal cores. In the case of the C 1s region, the following three main binding energy components were identified: C-C and C-H bindings at 284.8 eV; C-OH and C-N bindings at 286.3 eV; and C=O and C=N bindings at 287.8 eV [34], which clearly refer to the presence of the thiamine molecule on the NCs’ surface. For the S 2p region, the 2p_3/2_ transition at 164.6 eV might be assigned to the sulfur atoms in the thiazole aromatic ring of Vitamin B_1_. In the case of the N 1s region, four different types of nitrogen were detected: pyrimidine N at 398.7 eV, the amino group at 400.0 eV, the larger amount of thiazone-N at 401.0 eV, and some oxidized nitrogen at the 406.2 eV peak position [35]. While the stabilizing ligand was applied in the form of hydrochloride, some chloride contaminations were also discovered. Finally, the signal from the oxygen content originated mainly from the -OH form (531.7 eV) and a small amount of adsorbed H_2_O (533.4 eV).

To support the coordination of N-donor(s) to the metal cores, the IR spectra of the purified NCs in powder form were registered. Based on the registered FT-IR spectra (Figure S3), it can be concluded that the vibration of the primer -NH_2_ stretching at 3426 cm^−1^, the aromatic C=N in the pyrimidine ring at 1658 cm^−1^ and 1600 cm^−1^, and the characteristic vibrations of the thiazole rings in the far-middle IR region between 1000–500 cm^−1^ have shifted [36,37]. It should be noted that due to the gold reduction, some oxidized thiochrome forms have spontaneously synthesized. Therefore, it is complicated to clearly identify the vibration bands on the IR spectra. However, the primer coordination via pyrimidin-N1 can be assumed based on the crystallographic data of similar soft metal ion-thiamine complexes [38,39,40]. Moreover, the dominant change of the intensities between 3500 and 3000 cm^−1^ was also observed thanks to the presence of the metallic surface [41,42].

We evaluated the stability of the prepared NCs as well. The pH-dependent DLS and fluorescence measurements (Figure S4) were carried out, which can be used to determine the stability region of the B_1_-Au NCs. Note that the primer sizes of the cluster cores cannot be directly observed due to their small sizes. However, the cluster adducts in aqueous media are measurable with classical colloidal techniques. Based on the registered data, it can be concluded that the prepared NCs show a weak fluorescence under acidic conditions due to the large charge-shielding effect of the environment and form smaller aggregates based on the measured hydrodynamic diameters (d_H_ ~ 15 nm). In contrast, a dominant aggregation process can be observed at pH = 6.0 because the detected d_H_ is ca. 630 nm, and the ζ-potential is also close to zero mV. A possible explanation is that, at this pH value, the dominant form of the free thiamine is the pseudobase form [43]; thus, the sterically stabilizing molecules lost their dominant charge excess, and the formed NCs’ structure becomes unstable. Nonetheless, the fluorescent intensity shows a growing tendency due aggregation-induced emissions (AIE), in which intramolecular rotations and vibration are inhibited due to the close contact of cluster cores [44]. The alkaline condition has no dominant effect on the sizes and the PL property; the detected d_H_ values are close to the lower limit of the nanometer range. The stability is also proven by the measured ζ-potential values (|ζ| > 20 mV).

### 3.2. Antioxidant Studies

It is well known that the Vitamin B molecular family shows an excellent antioxidant effect [45]. In our previous study, it was shown that the Vitamin B_3_ derivative niacinamide, after the synthesis of Au NCs, is still usable as an antioxidant agent (ORAC value for B_3_ (amide) = 0.56 µM, for B_3_ (amide)-Au NCs = 0.48 µM) [46]. Thiamine shows a larger preventive effect against ROS with a chain breaker mechanism [47], because the antioxidant can donate an electron and a proton to ROS at the same time, which annihilates the harmful effect of ROS on the chemical environment.

Apart from the surface ligand Vitamin B_1_, gold-based nanomaterials can contribute to this mechanism due to their electron-rich surfaces [48]. For the determination of the potential antioxidant activity of the B_1_-Au NCs, two different methods were selected, in which the concentration was related to the B_1_ content (determined by spectrophotometry measurements) in the samples.

**Figure 2:**
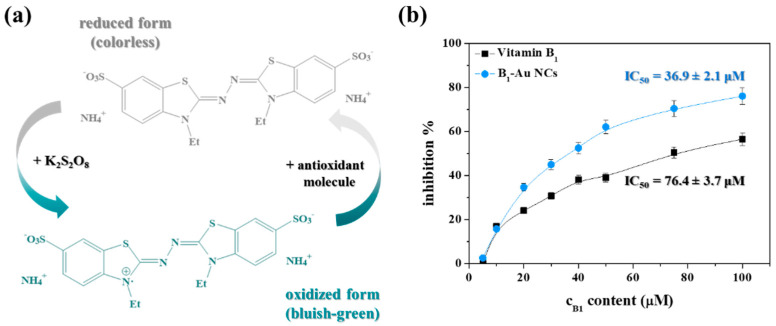
(**a**) The transformation of the ABTS molecule during the antioxidant measurement protocol from the reduced form to the oxidized form. (**b**) The radical scavenging activity curve (λ_abs_ = 734 nm, T = 25 °C) of the pure Vitamin B_1_ (■) and the B_1_-Au NCs (●) applying 5–100 μM B_1_ content.

In the first case, the ABTS test was applied, in which the fading of the bluish-green color at 734 nm was registered with a spectrophotometer (Figure 2a). This method has several advantages; it can be used for both hydrophilic and lipophilic compounds, it can be applied in a wide pH range, it is cheap, and the total antioxidant capacity of a complex sample (e.g., nanoclusters, metal alloys, other nanohybrid systems) can be determined. For the measurement, the pure Vitamin B_1_ served as a reference. As Figure 2b shows, the inhibition percent strongly depends on the applied concentration (In the case of the B_1_-Au NCs, the concentration was related to the Vitamin B_1_ content of the NCs suspension). In the case of the pure B_1_, the IC_50_ value was determined to be 76.4 ± 3.7 μM. In contrast, the presence of the Au cores, as well as the oxidized thiochrome in the cluster surface, can facilitate scavenging activity, because the IC_50_ value is 36.9 ± 2.1 μM, which clearly demonstrates the better antioxidant activity of the prepared complex structure than that of the initial pure thiamine.

**Figure 3:**
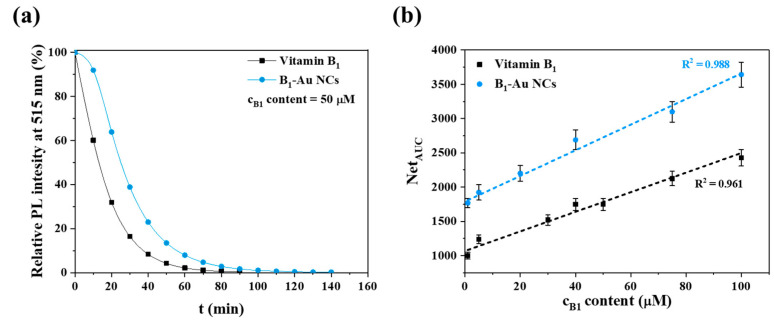
(**a**) The representative kinetic curves of fluorescence decay during the ORAC test in the presence of Vitamin B_1_ (■) and B_1_-Au NCs (●) (B_1_ content of 50 μM, T = 25 °C, λ_ex_ = 485 nm). (**b**) The antioxidant dose-response curves between 1 and 100 μM of B_1_ content.

To investigate the radical chain reaction, an ORAC assay was applied, in which the inhibition of the oxidation of peroxyl radicals was studied. For the measurement in an aqueous medium, the hydrophilic 2,2′-azobis(2-amidinopropane) dihydrochloride was selected as the ROS generator for the oxidative degradation of the fluorescein molecule. As Figure 3 shows, the ORAC assay also demonstrates the better antioxidant activity of the newly prepared B_1_-Au NCs. Based on the calculations, it can be estimated that the ORAC values are 0.36 ± 0.02 μM and 0.53 ± 0.01 μM in the case of Vitamin B_1_ and B_1_-Au NCs, respectively. As the calculated values demonstrate, both methods were suitable to determine the enhanced antioxidant behavior of the newly prepared blue-emitting Au NCs compared to that of the pure Vitamin B_1_.

### 3.3. Interaction of B_1_-Au NCs with Bovine Serum Albumin

For future biomedical applications as in vitro or in vivo antioxidant agents, it is necessary to examine the interaction of the B_1_-Au NCs with biologically important macromolecules. For this purpose, bovine serum albumin (BSA) was chosen as a model protein. Firstly, CD and PL measurements were carried out to analyze the optical characteristics of the interaction. During these measurements, the concentration of clusters was based on the metal content. The dispersion containing the NCs was prepared from their lyophilized powder form to obtain the exact metal concentration. During the registration of the CD spectra, the concentration of the BSA was constant (c_BSA_ = 0.05 mM), while the amount of the NCs varied between 0, 0.05, 0.01, 0.1, and 0.5 mM in the individual samples. The final samples were diluted 600-fold to reach the suitable optical signal. The spectra are presented in Figure S5, which show that the increasing cluster concentration had an increasing effect on the α-helix ratio from 54.3% to 73.2%. Therefore, it can be assumed that the binding process caused a contraction in the whole protein chain, which promoted the formation of an ordered structure.

**Figure 4:**
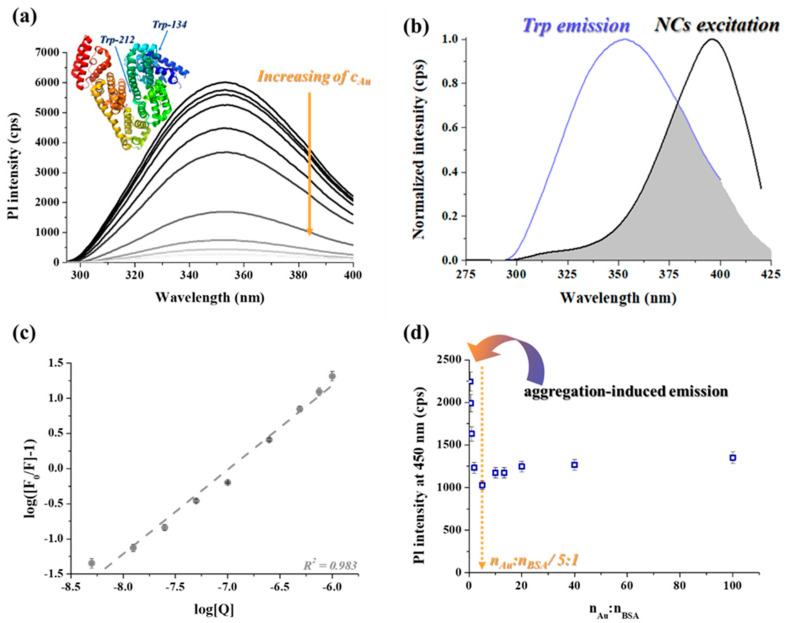
(**a**) The emission spectra of the BSA after addition of 0–1 mM Au NCs with the structure of BSA (PDB code: 3V03) (c_BSA_ = 0.5 mM; T = 25 °C; λ_ex_ = 280 nm). (**b**) The normalized emission spectrum of the Trp in BSA and the excitation spectrum of the B_1_-Au NCs. (**c**) The Scatchard plot of the experienced quenching at 350 nm. (**d**) The change in the PL intensity of B_1_-Au NCs depending on the amount of BSA (c_Au_ = 0.5 mM; λ_ex_ = 395 nm; λ_em_ = 450 nm; T = 25 °C).

To further investigate this binding process, PL measurements were carried out in two ways. In the first case, the quenching of the *Trp* amino acids of the protein chain in the 134th and 212th positions was examined. The fluorescence of this amino acid strongly depends on the chemical environment; thus, the conformation change of the protein chain also has a dominant impact on the extent of the intensity or the position of the maximum value. During these PL studies, the protein concentration was fixed at 0.5 mM. As it can be seen in Figure 4a, the intensity of the Trp emission decreases with the increase in the cluster’s concentration. Based on the standard Stern–Volmer evaluation of the data (Figure S6), in which the relative fluorescence (F_0_/F) is calculated depending on the applied Au concentration, it can be concluded that the binding causes a dominant quenching for the Trp emission. This decreasing tendency cannot be defined purely as static or dynamic quenching due to the upward curvature of the plot. Based on this phenomenon, the formation of a dark complex (static quenching) and an energy loss by collision (dynamic quenching) can also be present at the same time owing to the binding process. Moreover, the filling of the non-accessible binding sites, therefore the subsequent exposure of the Trp residues to the aqueous environment due to the change of the protein folding, can also cause similar differences from the linear Stern–Volmer-based dependence [49]. To further investigate the quenching process and the optical characteristics of the partners, the possibility of a resonance energy transfer (RET) between the BSA and B_1_-Au NCs should also be considered due to the large overlap of the emission and excitation spectra of the Trp and B_1_-Au NCs [50]. During this process, the energy excess can transfer from the donor (Trp in BSA) to an acceptor (Au NCs) via dipole–dipole coupling (Figure 4b), which can be facilitated by the bonding of the ultra-small cluster structures along the protein chain. This RET between the protein and NCs can also cause the nonlinearity of the quenching curves.

For this reason, the evaluation of the optical measurements was re-assessed based on the Scatchard plot (Equation (3)), which is a special methodology for protein/small molecule or protein/small particle interactions [51]:

(3) $$\log\left(\frac{F_{0}}{F}-1\right)=\log K_{a}+N\log\left[Q\right]$$

where the *F*_0_ and *F* are the emission intensities at 350 nm before and after the addition of NCs, respectively, *K_a_* is the binding constant, *N* is the number of binding sites of BSA to the cluster, and [*Q*] is the applied NC concentration [52].

By linear fitting (Figure 4c) the data points, the calculated *K_a_* value is 2.34 × 108 ± 1.17 × 107 M^−1^, which indicates a stronger interaction between the protein and NCs [53,54]. The determined *N* is 1.20 ± 0.05, which indicates a self-catalyzed binding; therefore, the affinity continually increases with the adsorption [53]. Based on the calculated *N* value, the presence of the metal core can slightly inhibit the attachment of thiamine in the binding pockets (Sudlow sites) of the BSA. Based on the $\Delta G=-RT\ln K_{a}$ equation, where *R* and *T* are the gas constant and the measurement temperature, respectively, the calculated Gibbs energy change of the interaction is −47.8 ± 2.3 kJ·mol^−1^, which indicates a spontaneous, thermodynamic favorable binding process. Nonetheless, the internal and external surfaces of the protein molecule are also suitable to adsorb the clusters by hydrophobic and electrostatic interactions via surface ligands, as well as the emergence a strong Au–S bond via cysteine amino acids in contrast with the pure molecule. The possible H-bond and hydrophobic interactions between Vitamin B_1_ and BSA are supported based on the work of R. Hosseinzadeh and K. Khorsandi, which included potentiometric and molecular docking studies [55]. Furthermore, in the work of M. Mallappa [56], spectroscopic and theoretical studies of similar bindings were also presented. Based on their measurements, it was concluded that the number of the binding sites is ca. 0.9, independently of the studied temperature, and the binding process is thermodynamically favorable (ΔG ~ −22 kJ mol^−1^). Additionally, the docking analysis showed that the binding is based on predominantly hydrophobic and H-bond interactions. Compared with the preciously published data, it can be clearly concluded that the presence of the ultra-small gold cores can facilitate the binding between the B_1_ and BSA considering the determined number of binding sites, but the interaction is still spontaneous and predominantly driven by hydrophobicforces. However, this phenomenon should be further investigated by other techniques.

Besides the *Trp* quenching, the change in the NCs’ emission by varying the BSA ratio was also investigated. Figure 4d shows that the intensity values interestingly reach a local minimum at a NCs:BSA/5:1 molar ratio. By applying a high cluster excess with large n_Au_:n_BSA_ ratios, the detected reduced fluorescence intensities are almost constant. In contrast, the use of a large BSA concentration (at small n_Au_:n_BSA_ ratios) can cause a dominant AIE effect due to the formation of dimers and tetramers of BSA [57], in which the bonded clusters in the folded proteins can repeatedly come close to each other, facilitating the energy transfer between the individual cluster cores. To verify the calculated *K_a_* and Δ*G* values of the protein/cluster interaction, calorimetry measurements were carried out, in which the RET cannot influence the detected quenching process and it is not able to distort the calculated values.

**Figure 5:**
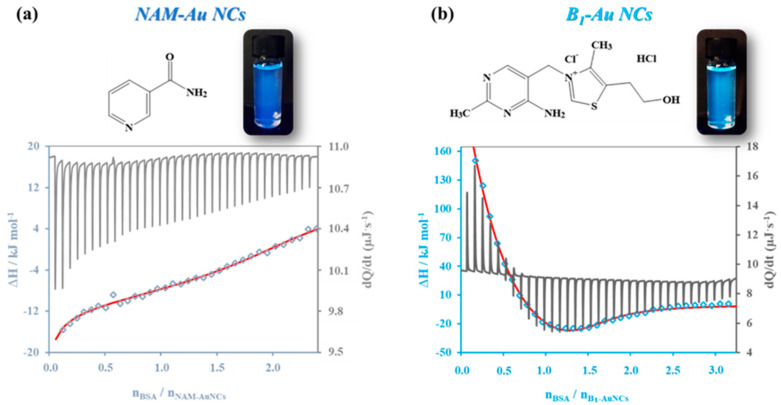
The calorimetric enthalpograms of the interaction between BSA and (**a**) NAM-Au NCs or (**b**) B_1_-Au NCs’ fitting by two-site binding model with the structural formula of the stabilizing ligands and the photos of the NC samples under UV lamp (λ_max_ = 365 nm).

The confirmation of the potential bioactivity was also investigated by comparisons with previously prepared antioxidant niacinamide-stabilized Au NCs (NAM-Au NCs) [46]. As can be seen on Figure 5, the interaction with the serum protein dramatically depends on the chemical structure of the stabilizing molecule of the metal surface. In the case of the NAM-Au NCs, the NAM molecule has only a few functional groups, which are mostly occupied by the Au NCs. The possible negligible binding by the BSA can be decisively facilitated by hydrophobic interactions via the aromatic ring of NAM. Moreover, the prepared metal cores are significantly smaller than in the case of the B_1_-Au NCs. Based on the registered enthalpograms, it can be concluded that the NAM-Au NCs do not show any considerable interaction with BSA. In contrast, the characteristic *K_a_* value of the B_1_-Au NCs/BSA complex systems, as well as their thermodynamic nature and stoichiometry, can be determined by the application of a two-site binding model with best fitting.

As can be seen in Table 1, the assessed *K_a_* values are significantly lower than in the case of the optical measurement. According to the reciprocal values of *K_a_*, the dissociation constants are in the micromolar range, which are ca. 1.6 μM and ca. 6.2 μM in the case of the *N*_1_ and *N*_2_ sites, respectively. The magnitude of the dissociation constants refers to a medium-strength interaction between the protein and NCs. Furthermore, the binding is spontaneous and thermodynamically favorable (Δ*G* < 0) in the case of both sites. Nevertheless, the determined data indicate very different behaviors for the two binding pockets. In the case of the second site, the calculated stoichiometry (*N*_2_ ~ 1.30) is in good agreement with the evaluated fluorescence data (*N* ~ 1.20). The negative values of the Δ*H* and Δ*S* refer to the dominant presence of secondary forces, such as H-bonds and electrostatic interactions, during the formation of the BSA/NCs complex [58].

In contrast, in the case of the first site, the binding is negligible based on the calculated *N*_1_ value. Moreover, the binding is controlled by hydrophobic forces due to the positive values of Δ*H* and Δ*S* [59], which are in good agreement with the pure vitamin B_1_ and BSA interaction studies. Therefore, it can be concluded that the binding process predominantly depends on the surface Vitamin B_1_ molecules, but the presence of gold cores can facilitate this reaction.

## 4. Conclusions

In our article, the optical and structural characteristics of newly prepared B_1_-Au NCs are presented. Based on their intensive blue emissions, the Au_8_ cluster core presumably can utilize the excitation light with ca. 3%. The stabilization of the cluster cores is realized through the pyrimidine ring of the Vitamin B_1_ molecule; moreover, the oxidized thiochrome form also takes part in the final structure stabilization presumably by *π···π* stacking with surface ligands. Based on the ABTS and ORAC assays, it was estimated that the presence of the metal cores having few atoms can enhance the antioxidant capacity compared with that of pure thiamine. For the investigation of further bioactivity, the interaction with the BSA protein was also measured with three different techniques. Based on the measurement data, it was concluded that the binding of the clusters along the protein chain is self-catalyzed, and the interaction is spontaneous and mostly controlled by hydrogen bonds and electrostatic interactions.

## Figures and Tables

**Table 1:** The calculated binding constants (K_a_), the Gibbs energy (ΔG), the enthalpy (ΔH), and entropy changes (ΔS) with the stoichiometry (N) of the binding between BSA and B_1_-Au NCs based on the ITC measurement.

	First Binding Site	Second Binding Site
K_a_ (M^−1^)	6.1 × 10^5^ ± 8.4 × 10^4^	1.6 × 10^5^ ± 2.4 × 10^4^
ΔG (kJ·mol^−1^)	−32.99 ± 0.34	−29.73 ± 0.36
ΔH (kJ·mol^−1^)	779.90 ± 23.64	−97.36 ± 4.14
ΔS (J·mol^−1^·K^−1^)	2728 ± 0.00	−227 ± 0.00
N	0.20 ± 0.00	1.30 ± 0.00

## Data Availability

Data is contained within the article and supplementary material.

## Supplementary Materials

The following supporting information can be downloaded at: https://www.mdpi.com/article/10.3390/antiox12040874/s1. Figure S1: The measured PL intensities of individual samples depending on the examined parameters (λ_em_ = 450 nm, λ_ex_ = 395 nm). The optimized parameters are as follows: (a) applied Vitamin B_1_ concentration: c_Au_ = 1 mM; non-regulated pH; 20 °C; 1 day (b) the concentration of gold(III) salt: c_B1_ = 25 mM; pH = 3.5; 20 °C; 1 day (c) the pH of the reaction mixture: c_B1_ = 25 mM, c_Au_ = 1 mM; 20 °C; 1 day with the chemical structure of the thiamine forms (d) the temperature: c_B1_ = 25 mM; c_Au_ = 1 mM; pH = 3.5; 1 day (e) the time of the synthesis: c_B1_ = 25 mM; c_Au_ = 1 mM; pH = 3.5; 25 °C. Scheme S1: The schematic illustration of the B_1_-Au NCs formation with a possible final cluster structure (unscaled). Figure S2: The XPS spectra of the (a) carbon (C), the (b) sulfur (S), the (c) nitrogen (N), the (d) chlorine (Cl), and the (e) oxygen (O) content in the B_1_-Au NCs sample. Figure S3: The FT-IR spectra of Vitamin B_1_ (black) and B_1_-Au NCs (blue) in the range of 3500–1000 cm^−1^ (left) and 1000–500 cm^−1^ (right). Figure S4: The measured (a) hydrodynamic diameters (**○**) and the ζ-potential (■) values, as well as the (b) PL intensities of the B_1_-Au NCs depending on the pH. Figure S5: The CD spectra of the BSA after addition of B_1_-Au NCs in various concentrations (c_BSA_ = 0.083 μM). Figure S6: The Stern–Volmer plot of the *Trp* quenching in BSA by B_1_-Au NCs (c_BSA_ = 0.05 mM). Theoretical background for the ITC measurements [60].
