# Supplementary material for: Promising Bioactivity of Vitamin B1-Au Nanocluster: Structure, Enhanced Antioxidant Behavior, and Serum Protein Interaction
Source: Antioxidants (Basel). 2023 Apr 3;12(4):874. doi: 10.3390/antiox12040874 (PMC10135240; doi:10.3390/antiox12040874)
Supplement: Supplementary file 1 [file antioxidants-12-00874-s001.zip › antioxidants-2267007-supplementary.pdf]

SUPPORTING INFORMATION

Promising bioactivity of Vitamin B_1-Au nanoclusters. Structure, enhanced antioxidant behavior and serum protein interaction

Ditta Ungor [a,b,*], Gyöngyi Gombár [a], Ádám Juhász [a,b], Gergely F. Samu [b], Edit Csapó [a,b,*]

[a] MTA-SZTE Lendület "Momentum" Noble Metal Nanostructures Research Group, University of Szeged, Rerrich B. sqr. 1, H-6720 Szeged, Hungary

[b] Interdisciplinary Excellence Center, Department of Physical Chemistry and Materials Science, University of Szeged, Rerrich B. sqr. 1, H-6720 Szeged, Hungary

[*]Correspondence: ungord@chem.u-szeged.hu (D. Ungor) and juhaszne@chem.u-szeged.hu (E. Csapó)

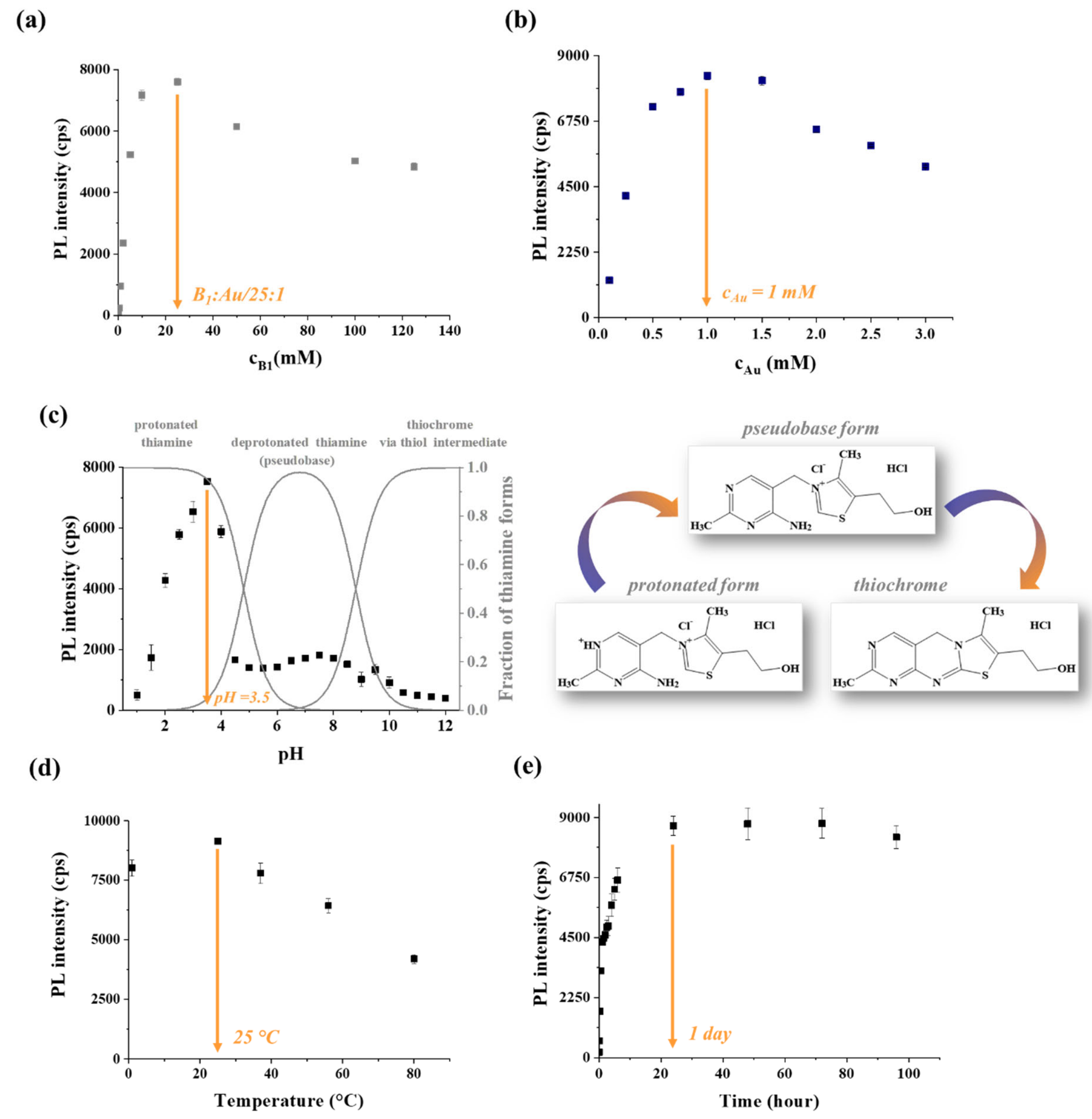

Figure S1. The measured PL intensities of individual samples depending on the examined parameters. (λ_{em} = 450 nm, λ_{ex} = 395 nm) The optimized parameters are: **(a)** applied Vitamin B_1 concentration: c_{Au} = 1 mM; non-regulated pH; 20 °C; 1 day **(b)** the concentration of gold(III) salt: c_{B1} = 25 mM; pH = 3.5; 20 °C; 1 day **(c)** the pH of the reaction mixture: c_{B1} = 25 mM, c_{Au} = 1 mM; 20 °C; 1 day with the chemical structure of the thiamine forms **(d)** the temperature: c_{B1} = 25 mM; c_{Au} = 1 mM; pH = 3.5; 1 day **(e)** the time of the synthesis: c_{B1} = 25 mM; c_{Au} = 1 mM; pH = 3.5; 25 °C

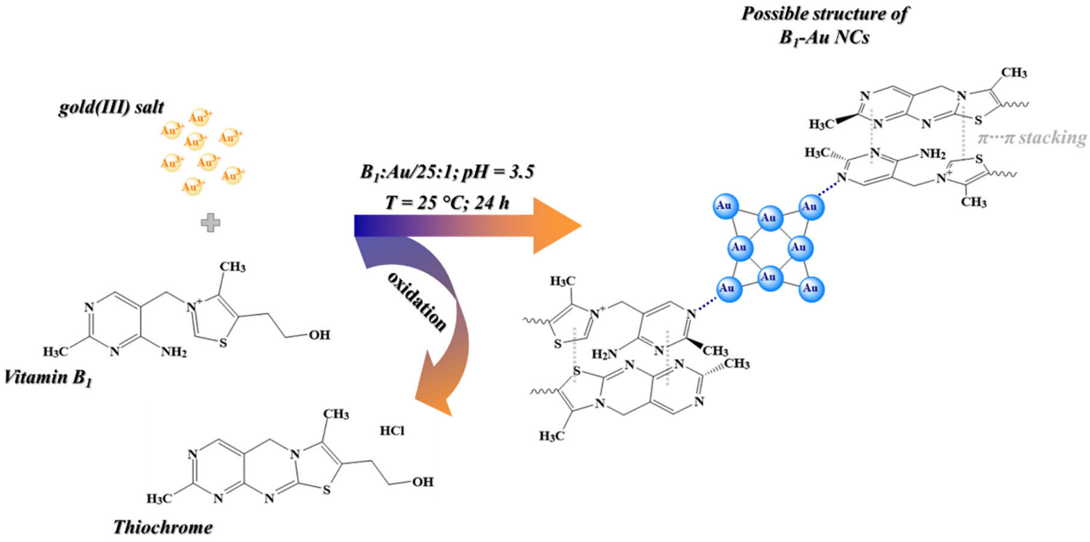

Scheme S1. The schematic illustration of the B_1-Au NCs formation with a possible final cluster structure (unscaled).

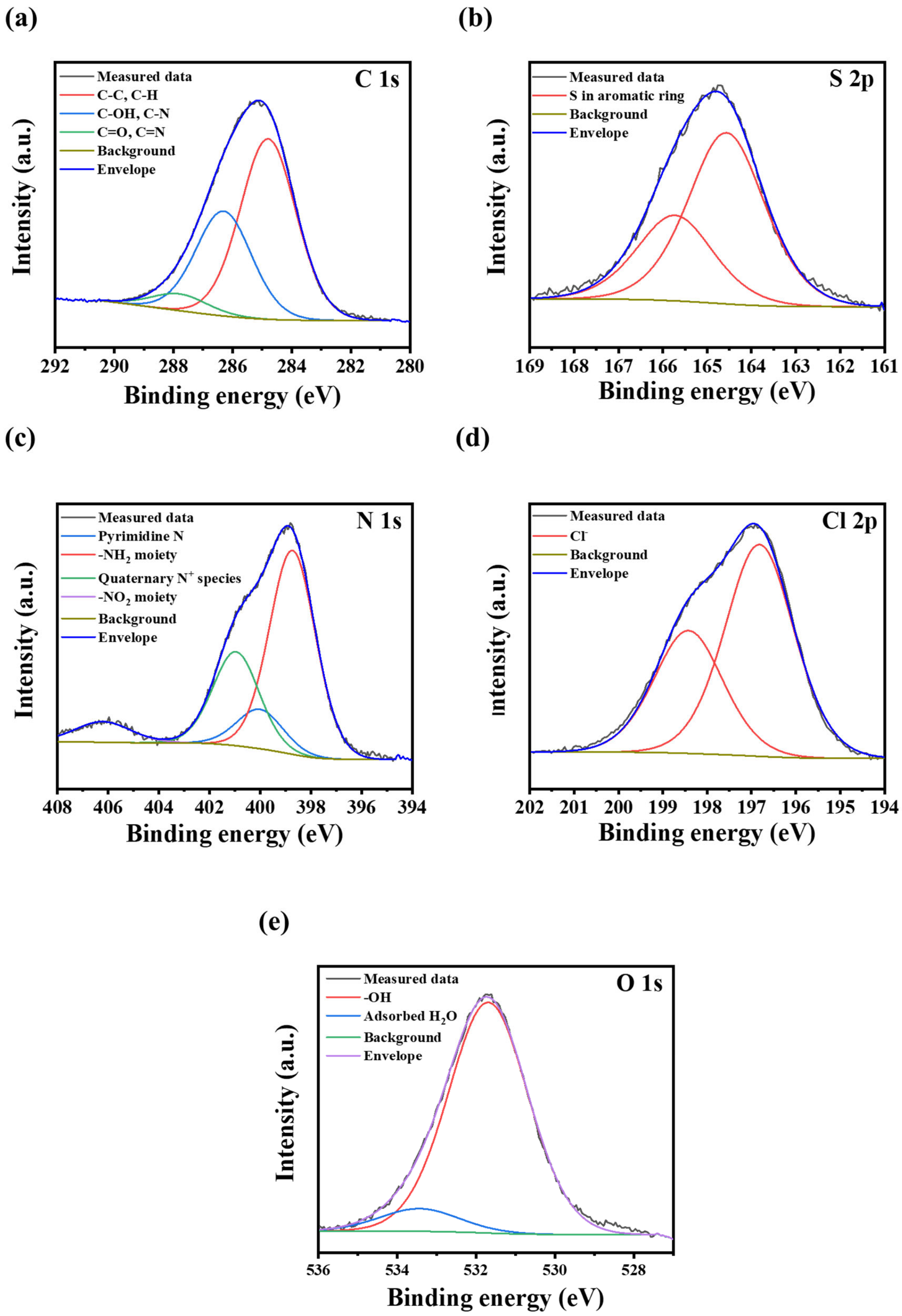

Figure S2. The XPS spectra of the **(a)** carbon (C) the **(b)** sulfur (S), the **(c)** nitrogen (N), the **(d)** chlorine (Cl) and the **(e)** oxygen (O) content in the B_1-Au NCs sample.

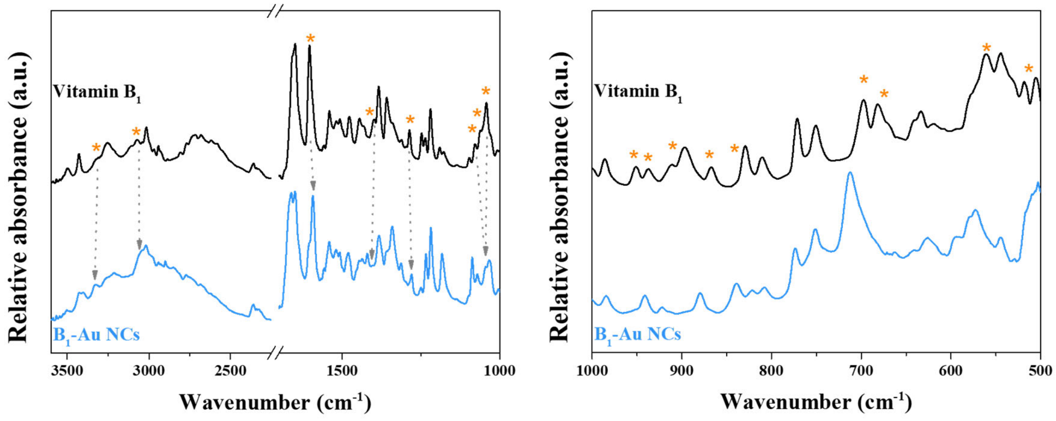

Figure S3. The FT-IR spectra of Vitamin B_1 (**black**) and B_1-Au NCs (**blue**) in the range of 3500 -1000 cm^{-1} (left) and 1000 -500 cm^{-1} (right).

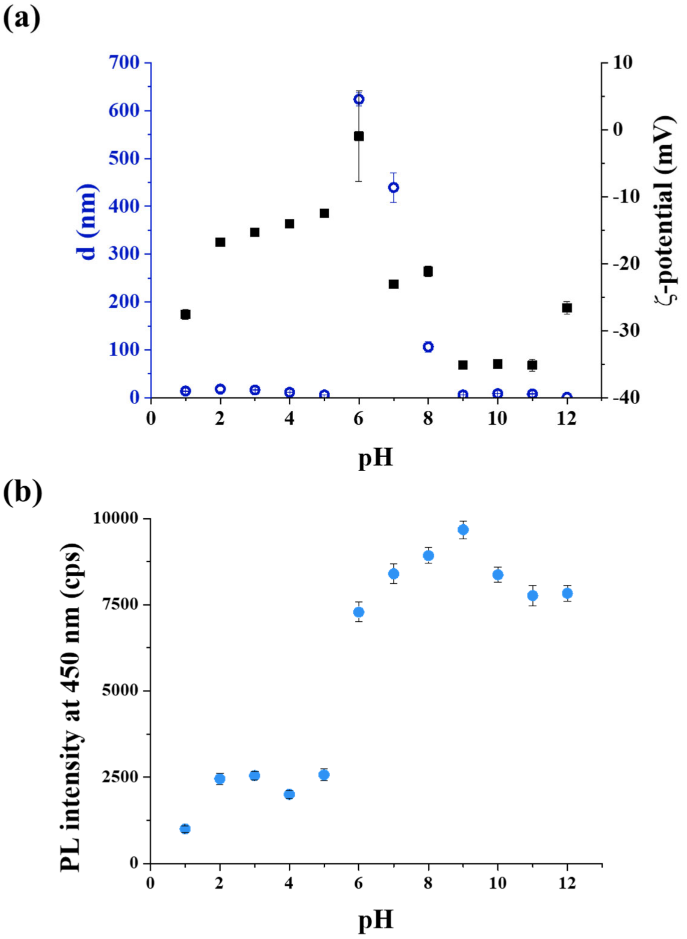

Figure S4. The measured **(a)** hydrodynamic diameters (○) and the ζ-potential (■) values, as well as the **(b)** PL intensities of the B_1-Au NCs depending on the pH.

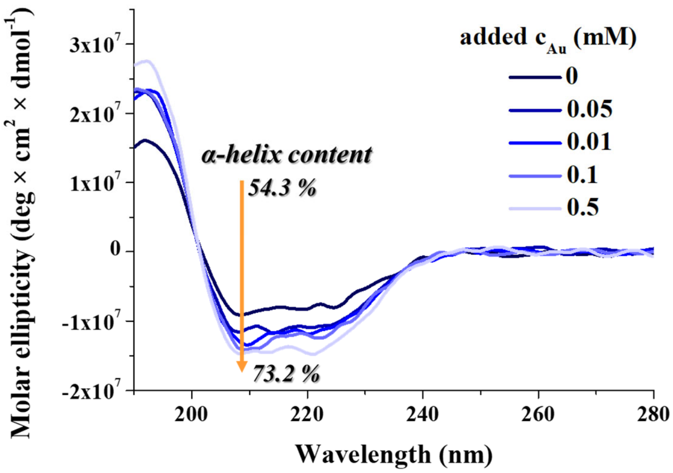

Figure S5. The CD spectra of the BSA after addition of B1-Au NCs in various concentration (c_{BSA} = 0.083 μM).

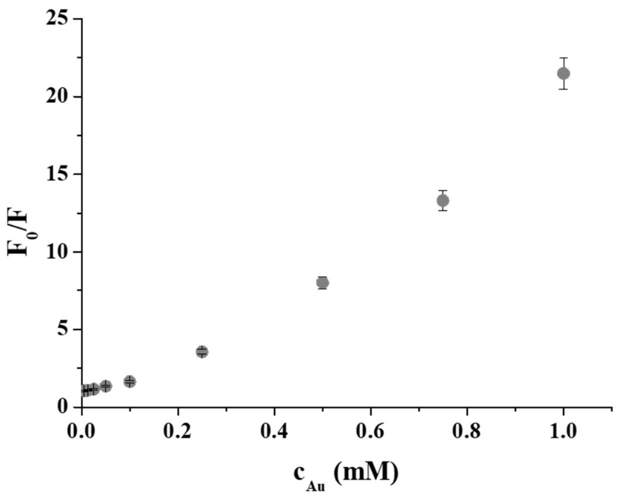

Figure S6. The Stern-Volmer plot of the *Trp* quenching in BSA by B_1-Au NCs (c_{BSA} = 0.05 mM).

Theoretical background for the ITC measurements

During the analysis of the data obtained from the reported calorimetric titrations were analyzed using the Origin ITC data analysis software (ITC Data Analysis in OriginH, **1998**, Tutorial Guide Version 5.0) supplied by the instrument manufacturer [1]. Data obtained for the interaction of BSA with BSA and NAM-Au NCs or B_1-Au NCs could be fit approximately using the 'one set of sites' binding model, whereas the binding data obtained for the interaction could be fit satisfactorily with the 'two sets of sites' binding model. These two models are briefly described below.

For ***one set of sites binding model*** the binding constant (***K***) and total ligand concentration (X_t) can be expressed according to the following equations,

$$K = \frac{\Theta}{(1-\Theta)[X]} \qquad (1)$$

$$X_t = [X]N\Theta M_t \qquad (2)$$

where, ***N*** is the number of the binding sites, V_o is the active volume of the cell, M_t and ***[M]*** are bulk and free concentration of macromolecule in the V_o volume, while X_t and ***[X]*** are bulk and free concentration of ligand, and ***Θ*** is the fraction of sites occupied by ligand X. Combining Eq. (1) and Eq. (2) gives a quadratic equation form:

$$\Theta^2 - \Theta\left[1 + \frac{X_t}{NM_t} + \frac{1}{NKM_t}\right] + \frac{X_t}{NM_t} = 0 \qquad (3)$$

The total heat content **Q** of the solution contained in V_o (determined relative to zero for the unliganded species) at fractional saturation ***Θ*** is

$$Q = N\Theta M_t \Delta H V_0 \qquad (4)$$

where ***ΔH*** is the molar heat of ligand binding. Solving the quadratic Eq. (3) for ***Θ*** and then substituting this into Eq. (4) gives

$$Q = \frac{NM_t\Delta HV}{2}\left\{1 + \frac{X_t}{nN} + \frac{1}{NKM_t} - \left[\left(1 + \frac{X_t}{NM_t} + \frac{1}{NKM_t}\right)^2 - \frac{4X_t}{NM_t}\right]^{\frac{1}{2}}\right\} \qquad (5)$$

In this way, for a system of one set of identical binding sites, the total heat evolved (or absorbed) during the binding process at the end of the ith injection, ***Q(i)***, is given by Eq. (5) where ***N*** is the number of binding sites, M_t is the total protein concentration, X_t is the total ligand concentration, ***V*** is the cell volume, ***K*** is the binding constant and ***ΔH*** is the binding enthalpy.

The value of ***Q*** above can be calculated (for any designated values of ***N***, ***K***, and ***ΔH***) at the end of the ***i***th injection and designated ***Q(i)***. The parameter of interest for comparison with experiment, however, is the change in heat content from the completion of the ***i-1*** injection to completion of the ***i*** injection. The expression for ***Q*** in Eq. (5) only applies to the liquid contained in volume V_o. Therefore, after completing an injection, it is obvious that a correction must be made for displaced volume (*i.e.*, ΔV_i = injection volume) since some of the liquid in V_o after the ***i-1*** injection will no longer be in V_o after the ***i***th injection, even though it will contribute to the heat effect (assuming the kinetics of reaction and mixing are fast) before it passes out of the working volume V_o. The liquid in the displaced volume contributes about 50% as much heat effect as an equivalent volume remaining in V_o[1].The heat corresponding to the ***i***th injection ***ΔQ(i)***, is equal to the difference between ***Q(i)*** and ***Q(i-1)*** and is given by Eq. (6), which involves the necessary correction factor for the displaced volume (the injection volume ***dVi***):

$$\Delta Q(i) = Q(i)\ \frac{dV_i}{V_0}\left[\frac{Q(i)-Q(i-1)}{2}\right] - Q(i-1) \quad (6)$$

The ITC unit measures ***ΔQ(i)*** value for each injection. These values are then fitted to Eq. (1) and (2) by a nonlinear least squares method. The fit process involves initial guess of ***N***, ***K*** and ***ΔH*** which allows calculation of ***ΔQ(i)*** values as mentioned above for all injections and comparing them with the corresponding experimentally determined values. Based on this comparison the initial guess of ***N***, ***K*** and ***ΔH*** is improved and the process is repeated till no further significant improvement in the fit can be obtained.

For a system with ***two sets of independent binding sites***, using the same definition of symbols as above for set 1 and set 2, we have

$$K_1 = \frac{\Theta_1}{(1-\Theta_1)[X]} \quad K_2 = \frac{\Theta_2}{(1-\Theta_2)[X]} \qquad (7)$$

While the total ligand concentration is given by Eq. (5)

$$X_t = [X] + M_t(N_1\Theta_1 + N_2\Theta_2) \qquad (8)$$

Solving Eq. (7) for Θ_1 and Θ_2 and then substituting into Eq. (8) gives

$$X_t = [X] + \frac{N_1 M_t [X] K_1}{1+[X]K_1} + \frac{N_2 M_t [X] K_2}{1+[X]K_2} \qquad (9)$$

Clearing Eq. (6) of fractions and collecting like terms leads to a cubic equation of the form

$$[X]^3 + p[X]^2 + q[X] = 0 \quad (10)$$

where

$$p = \frac{1}{K_1} + \frac{1}{K_2} + (N_1 + N_2)M_t - X_t \quad (11)$$

$$q = \left(\frac{1}{K_2} + \frac{1}{K_1}\right) M_t - \left(\frac{1}{K_1} + \frac{1}{K_2}\right) X_t + \frac{1}{K_1 K_2} \quad (12)$$

$$r = \frac{-X_t}{K_1 K_2} \quad (13)$$

Equations (10–13) can be solved for ***[X]*** either in closed form or (as done in Origin) numerically by using Newton's Method if parameters N_1, N_2, K_1, and K_2 are assigned. Both Θ_1 and Θ_2 may then be obtained from Eq. (7) above. As discussed earlier, the heat content after any injection ***i*** is equal to the total heat evolved (or absorbed) during the binding process at the end of the ith injection, ***Q(i)***, is given by Eq. (14):

$$Q = M_t V(N_1 \Theta_1 \Delta H_1 + N_2 \Theta_2 \Delta H_2) \quad (14)$$

where N_1 and N_2 are the number of binding sites of type 1 and type 2, and ΔH_1 and ΔH_2 are the corresponding enthalpies of binding. Θ_1 and Θ_2 are the fractions of the type 1 and type 2 sites that are occupied and are related to the association constants K_1 and K_2, according to Eq. (7). After a similar correction for displaced volume, the pertinent calculated heat effect for the ***i*** injection is equivalent the earlier described results in Eq. (6):

$$\Delta Q(i) = Q(i)\ \frac{dV_i}{V_0}\left[\frac{Q(i) - Q(i-1)}{2}\right] - Q(i-1) \quad (15)$$

which may be used in the Marquardt algorithm to obtain best values for the six fitting parameters (N_1, N_2, K_1, K_2, ΔH_1 and ΔH_2). The fit process involves initial guess of N_1, N_2, K_1, K_2, ΔH_1 and ΔH_2 which allows calculation of ***ΔQ(i)*** values as mentioned above for all injections and comparing them with the corresponding experimentally determined values. Based on this comparison the initial guess of the values of the above parameters is improved and the process is repeated till no further significant improvement in the fit can be obtained.

From the values of $\boldsymbol{K}$ and $\boldsymbol{\Delta H}$, the thermodynamic parameters, $\boldsymbol{\Delta G}$ and $\boldsymbol{\Delta S}$ are calculated according to the basic thermodynamic Equations (16) and (17):

$$\Delta G = -RT\ln K \qquad (16)$$

$$\Delta S = \frac{\Delta H - \Delta G}{T} \qquad (17)$$

[1] Wiseman, T.; Williston, S.; Brandts, JF.; Lin, L-N. Rapid measurement of binding constants and heats of binding using a new titration calorimeter. *Anal. Biochem.* **1989**, *179*, 131–137.
